# Extraction and Purification of Nicotine from Tobacco Rhizomes by Supercritical CO_2_

**DOI:** 10.3390/molecules29051147

**Published:** 2024-03-05

**Authors:** Fangyuan Zheng, Qishan Xie, Qingguang Ren, Jilie Kong

**Affiliations:** Department of Chemistry, Fudan University, Shanghai 200433, China; 23110220125@m.fudan.edu.cn (F.Z.); 19307110511@fudan.edu.cn (Q.X.); qgren@fudan.edu.cn (Q.R.)

**Keywords:** nicotine, tobacco rhizomes, supercritical carbon dioxide extraction

## Abstract

Currently, in the ongoing development of the tobacco industry, a large amount of tobacco rhizomes is discarded as waste. These wastes are usually disposed of through incineration or burial. However, these tobacco wastes still have some economic value. High-purity nicotine has a promising market outlook as the primary raw material for electronic cigarette liquid. Nicotine is not only found in tobacco leaves but also in the rhizomes of tobacco plants. This study presents a method for treating tobacco waste and extracting high-purity nicotine from it. After mixing the raw material powder and entrainer in specific ratios, as much of the nicotine in tobacco roots can be extracted as possible using supercritical carbon dioxide extraction. The effects of temperature, the ratio of the entrainer, and the volume fraction of ethanol in the entrainer on the nicotine yield in supercritical fluid extraction (SFE) at 25 MPa for 120 min were discussed. By using 90% ethanol (a raw material mass-to-volume ratio of 1:5) as the entrainer, we obtained the highest nicotine yield of 0.49% at 65 °C. Meanwhile, the purity of the crude extract was 61.71%, and after purification, it increased to 97.57%. In this way, we can not only obtain nicotine with market value but also further reduce the harm to the environment caused by tobacco waste disposal.

## 1. Introduction

Tobacco, one of the most widely grown non-food crops worldwide, generates enormous economic benefits. Tobacco leaves, serving as the primary raw material for cigarettes, possess significant value in the global market. Fragments or powder derived from tobacco leaves are commonly utilised for smoking and chewing. Nicotine, the principal alkaloid present in tobacco, is primarily responsible for cigarette addiction [1,2]. When smoking or using tobacco products, nicotine quickly enters the bloodstream through the lungs and then entering the brain, eventually resulting in addiction [2]. In recent years, nicotine has frequently been used as a replacement therapy in smoking cessation [3] and to ameliorate symptoms associated with Alzheimer’s disease [4]. Simultaneously, with the advancement of the electronic cigarette industry, this relatively novel method of nicotine delivery has garnered widespread attention among individuals. Each e-cigarette cartridge typically contains approximately 11.9 mg of nicotine [5]. By incorporating nicotine complexes into e-cigarette liquids, it becomes possible to facilitate smoking cessation [6] and mitigate both individual and public health risks associated with traditional cigarette consumption [7].

There is a large amount of tobacco waste generated annually during the industrial collection and processing of raw tobacco materials. This waste includes secondary tobacco leaves and tobacco rhizomes or branches [8]. More than 20% of raw tobacco materials are discarded as waste each year [9]. Due to their high content of total organic carbon and nicotine [10], these solid wastes cannot be disposed of through conventional landfills or burning methods [11]. Therefore, it is of significance to focus on reprocessing existing tobacco waste in order to obtain commercially valuable natural products and minimize tobacco waste’s environmental impact.

Generally, the nicotine content in tobacco leaves ranges from 2% to 6%. Tayoub et al. determined the nicotine content in the dry weight of five different types of tobacco leaves using HPLC and LS-MS techniques, yielding values ranging from approximately 3.3% to 6.7% [12]. In 2022, Djapic et al., extracted nicotine from two types of tobacco leaves individually and optimised the experimental conditions using the SC-CO_2_ extraction technique. Their extracted nicotine-to-raw material dry weight ratios of 2.33% and 2.99%, respectively [13]. This method can also be applied for treating secondary tobacco leaves found in tobacco waste as well as powdered leaves. For instance, J.RincoN et al. have attempted to treat leaf bits and powder present in tobacco waste using SC-CO_2_ technology [14]. Meanwhile, Purwono et al., in a previous report, measured the nicotine content in tobacco rhizomes via planar chromatography, which ranged between approximately 0.49% and 0.51% [15]. However, there are currently no relevant developments regarding the extraction of nicotine from tobacco rhizomes.

SC-CO_2_ technology involves utilizing carbon dioxide under supercritical fluid conditions as a solvent for target extraction by manipulating pressure and temperature parameters. After extraction is complete, the carbon dioxide evaporates back into its gaseous state again [16]. This method effectively minimizes the impact of solvents on the extraction process. The SC-CO_2_ extraction process involves various influencing factors such as temperature, time, and pressure. When extracting nicotine from tobacco rhizomes, it is also important to consider the polarity of nicotine and the use of entrainers. The objective of this research is to synthesize previous studies or reports in order to design extraction methods that facilitate efficient nicotine extraction from tobacco rhizomes. Additionally, the experimental conditions were optimised to increase the nicotine extraction rate and to establish a method for separating and purifying crude extracts to obtain high purity nicotine. In this study, we utilised Zimbabwean tobacco rhizomes, which consist of small branches and stems.

Conventional nicotine extraction methods involve the use of many hazardous chemicals, the separation of complexes, and cumbersome procedures [17,18]. There is a lot of unnecessary waste and consumption involved in this process [19]. SC-CO_2_ technology, as an emerging green extraction technology, has many advantages. Firstly, supercritical fluid CO_2_ is less viscous than conventional liquid solvents while offering better diffusion and conduction properties [20]. Secondly, due to the inert nature of supercritical CO_2_, we can obtain solvent-free targets after completing the extraction [21,22]. This technique makes the purification of crude extracts easy and reduces waste. Finally, the operation of supercritical extraction is simple and the parameters are easy to adjust [23]. Supercritical fluid extraction units are usually built in one piece, allowing for easy adjustment of temperature, extraction time, and other parameters. Fraction collection and instrument cleaning are conducted automatically [24]. At this stage, ton-scale supercritical CO_2_ extraction machines are already in operation. Given this progress, large-scale industrial production and waste treatment is feasible. The treatment of tobacco waste by SC-CO_2_ technology is of great significance for the development of the green tobacco market.

Based on the results of the above studies, further investigations were conducted regarding the characteristics of tobacco rhizomes and nicotine: (1) examining how temperature influences the rate of nicotine extraction from tobacco rhizomes; (2) analysing how varying ratios of entrainer to sample and volume fractions of ethanol in the entrainer affect the nicotine extraction rate; (3) conducting chemical spectrum analysis using GC-MS on the extracted samples; (4) determining the optimal extraction conditions based on orthogonal experiments considering all aforementioned influencing factors; and (5) purification at the laboratory level.

## 2. Results

**Figure 1:**
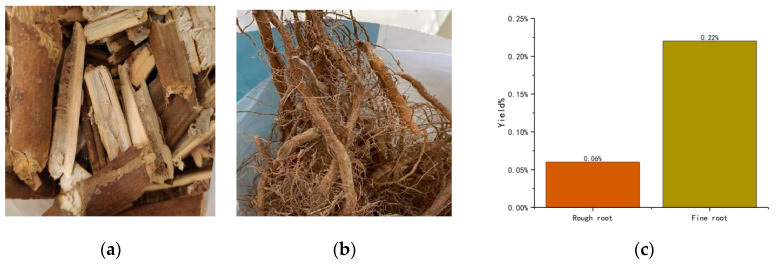
(**a**) Rough rhizomes; (**b**) fine rhizomes; (**c**) nicotine yield (%) of both rhizomes.

The rhizomes of tobacco can be broadly divided into two types. One is the main root of tobacco, which is close to or buried under the soil (Figure 1a). In appearance, it resembles firewood, with a hard texture. Its main component is plant cellulose. The other type consists of the side rhizomes or branches of tobacco, which are slenderer in shape (Figure 1b). These can be broken easily by hand. In this experiment, we ground each of these rhizomes into a powder. The nicotine was extracted by SC-CO_2_ without any other treatment. The yields of rough and fine rhizomes were 0.06% and 0.22% (Figure 1c), respectively. Apparently, the yield of nicotine in the rough rhizomes was not satisfactory. This may be due to the structure of the crude root not being favourable for extraction. In the meantime, the content of organic carbon such as cellulose in this rhizome type is higher for the same mass. Therefore, the main plant material we explored in this experiment consisted of fine rhizomes.

### 2.1. Factors Affecting Nicotine Yield

**Figure 2:**
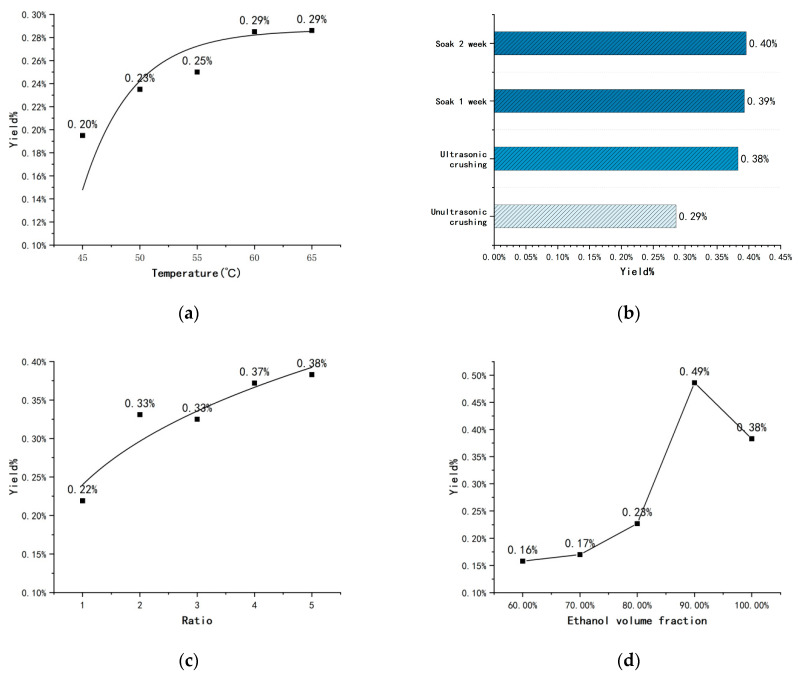
(**a**) Effect of temperature on the nicotine yield (%) of tobacco rhizomes; (**b**) effect of ultrasonic crushing and soaking on the nicotine yield (%) of tobacco rhizomes; (**c**) effect of the ratio of ethanol volume to the mass of materials on the nicotine yield (%) of tobacco rhizomes; (**d**) effect of ethanol volume fraction on the nicotine yield (%) of tobacco rhizomes.

Based on previous research, it can be roughly confirmed that nicotine yield is positively correlated with time and pressure [13,14,25,26]. The objective of this experiment is to establish optimal research conditions for maximum yield and facilitate potential industrial scale-up, taking into consideration equipment, safety, and manual operation. Therefore, after some pre-experimental verification, a longer extraction time of 120 min and a logical and ordinary extraction pressure of 25 MPa were selected for this study. Under the above conditions, the impact of temperature, the proportion of entrainer used, and the volume fraction of the ethanol used as an entrainer on the yield were investigated. Firstly, in order to show the direct effect of temperature on the yield, we did not ultrasonically crush the materials, but kept other conditions constant, as shown in Figure 2a. We can observe that the yield increases slowly with the increase in temperature. In the temperature range of the supercritical state of CO_2_, the effect of temperature on the yield gradually levelled off when the temperature increased to nearly 60 °C. This means that when the temperature reaches 65 °C, the degree to which nicotine solubility is affected by temperature reaches its limit. Thus, increasing the temperature alone can increase the yield by about 45%, from 0.20% to 0.29%.

Due to the very low content of nicotine in tobacco rhizomes and the fibrous walls of its plant cells being very well developed, we tried to improve the release of nicotine by using an ultrasonic cell crusher. As shown in Figure 2b, under the same temperature, the yield of nicotine increased by about one-third after ultrasonic crushing. The improvement in yield with the application of ultrasonic crushing was quite significant, improving by 0.38%. This means that in the cells of tobacco rhizomes, the cell wall and membrane hinder nicotine extraction. Meanwhile, traditional plant extraction was performed by soaking. We mixed the powder from tobacco rhizomes with anhydrous ethanol and soaked it for some time. Small improvements can be observed.

Although the advantage of SC-CO_2_ is that it minimises the effect of solvents on the target extracted substances, entrainers still have a role to play. The use of polar entrainers plays an important role in the extraction of alkaloids from tobacco or other plants [8,27,28] and the removal of impurities from coals, such as iron and calcium [29,30]. In order to maximise the yield of nicotine, ethanol was our choice of solvent after considering the toxicity, boiling point, ease of solvent separation, and the cost of various organic entrainers. We kept increasing the ratio of ethanol volume to the mass of tobacco powder materials until it reached 1:5, beyond which the increase in yield levelled off as the ratio went up (Figure 2c). In other words, 100 mL of ethanol is required for every 20 g of raw material. This may be because at low ratios, the entrainer could not fully infiltrate the tobacco rhizome powder, or the small amount of entrainer may not be sufficient to cause the complete release of nicotine from the cells. However, when we conducted our study on the effect of volume fraction of ethanol on nicotine yield (shown in Figure 2d), unexpected results were obtained. After we used 90% ethanol as an entrainer, the nicotine yield showed a more substantial increase. The yield of nicotine extraction from tobacco rhizomes using the SC-CO_2_ technique reached about 0.49%. From previous determinations of nicotine content in tobacco rhizomes, extraction rates of almost 99% have been achieved [15]. Compared to the original method, nicotine yield was increased by about 1.2 times. We can assume that because nicotine is a more polar organic molecule compared to ethanol, it is more soluble in water. The lower volume fraction of ethanol contains more water; water also has a higher boiling point, and in the SC-CO_2_ system, it is difficult for water to completely evaporate and condense back into the extraction kettle. The effect of lower volume fractions of ethanol seems to be very poor. However, the right amount of water increases the polarity of the entrainer and improves the solubility of nicotine in it.

### 2.2. Design and Analysis of Orthogonal Experiments

In order to better determine the effects of temperature, entrainer ratio, and volume fraction of ethanol used as the entrainer on the yield of nicotine from tobacco rhizomes extracted by SC-CO_2_, we designed an orthogonal experiment. Through this experiment and performing an extremum difference analysis, we can obtain the pairing of these three factors at the best level. This means we can determine the best experimental conditions. Table 1 shows the design of the orthogonal experiment for the yield of nicotine from fine tobacco rhizomes and three replicates of the central point, totalling 11 experiments.

The extremum difference analysis results for three factors at the three different level of the orthogonal experiments for the total nicotine extraction yield of fine tobacco rhizomes are depicted in Table 2. The table shows that the best matches among the three factors correspond to level 3, level 3, and level 2. This implies that the optimal experimental conditions postulated by the extremum difference analysis are that the most nicotine can be extracted by using 90% ethanol as an entrainer in a ratio of 1:5 at 65 °C.

### 2.3. Component Analysis of Crude Extracts and Purified Products

**Figure 3:**
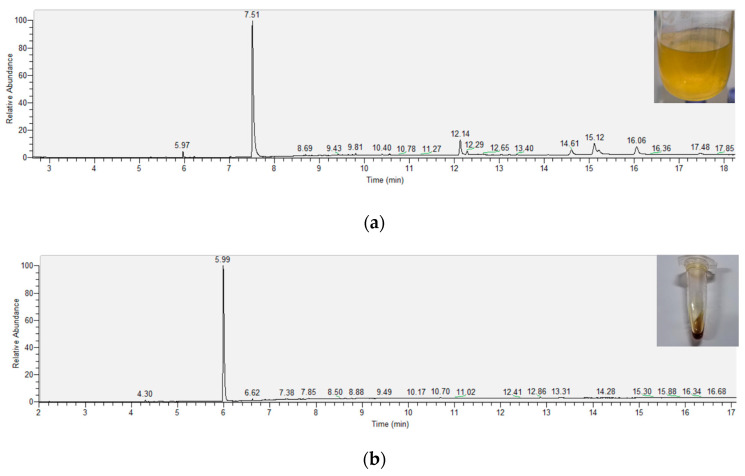
(**a**) GC-MS spectrogram of crude extracts of fine tobacco rhizomes; (**b**) GC-MS spectrogram of purified extract of fine tobacco rhizomes.

To further test the potential industrialisation of this experiment, we also attempted to purify the extracted crude product. The crude extract is a yellow-green suspension with some colourless floccules. In the crude extract of tobacco rhizomes, after removing ethanol as a solvent, we were able to calculate the relative content of nicotine to be approximately 61.71% from the peak area ratio of the GC-MS spectrogram (Figure 3a). From the spectrogram, we can notice that there are three distinct mass spectral peaks, indicating the presence of three impurities in significant quantities within the crude extract. The compound eluting at a retention time of 12.14 min corresponds to palmitic acid, constituting approximately 7.2% of the total content. The compounds with retention times of 15.12 and 16.04 min correspond to oleic acid and its derivatives, collectively accounting for around 20.39% of the relative content. At the laboratory level, we purified the extracts using column separation methods. After purification, the extract became a dark brown liquid. It was calculated that the purity of nicotine in the purified extracts from the tobacco rhizomes was about 97.57% based on the peak area ratio of the GC-MS mass spectrogram (Figure 3b). The results of mass spectrometry showed that most of the impurities in the tobacco rhizome extracts had higher boiling points than nicotine.

## 3. Discussion

Like tobacco, the main active ingredient in tobacco root waste is nicotine [31]. There are both rough and fine parts present in tobacco root waste. The rough root looks almost indistinguishable from ordinary firewood from the outside and has a hard texture. Experiments have shown that the coarse roots contain very little nicotine. This may lead to it being less harmful to the environment when subjected to burial and burning, but not having an economic value. Fine roots mainly come from tobacco crop branches, stems, and other parts which grow on the surface of the ground. Our experiments have been carried out using fine roots as the main object of study. The extraction rate of nicotine from fine tobacco rhizomes fluctuated between 0.16% and 0.49% under different pretreatment and extraction conditions. The highest extraction rate, 0.49%, was obtained by extracting tobacco rhizome powder dispersed with five times the volume of 90% ethanol at 65 °C and 25 MPa. Compared to tobacco leaves, which contain about 3% nicotine [13], Zimbabwean tobacco roots contain only one-sixth of the amount of nicotine found in tobacco leaves. Previous studies have shown that 50 °C is the optimal temperature for the extraction of nicotine from tobacco by SC-CO_2_ [13,32]. However, in this experiment, the nicotine yield did not stabilise until the temperature reached 60–65 °C. In plants, roots are usually stronger in structure than leaves. Therefore, higher temperatures are required when treating tobacco rhizomes.

In very early studies, Fischer found that adding an appropriate amount of methanol as a modifier to a system of SC-CO_2_ nicotine extraction increased the yield of nicotine [25]. This was thought to be due to methanol increasing the affinity of nicotine for CO_2_ at the time. Moreover, in the 2018 study by Wang et al., on the extraction of solanesol by SFE, a large amount of ethanol was used as an entrainer, which effectively increased the yield of solanesol [8]. Nerome et al. significantly improved the extraction rate of phytoactives from saffron using water and methanol as entrainers [33]. In this study, the effect of temperature and volume of entrainer used on nicotine yield agreed with previous studies. Furthermore, the effect of the ethanol volume fraction on nicotine yield was investigated. However, the study on this aspect produced some unexpected results. For example, the use of 90% ethanol showed a substantial improvement in the extraction. Conversely, if the volume fraction of ethanol continued to be reduced, the nicotine yield was significantly decreased. Nicotine is a pyridine cyclic organic compound, which leads to its higher polarity. We hypothesised that the polarity of the entrainer could be improved by adding a suitable amount of water to the ethanol, which would facilitate the dissolution of the nicotine molecules. Nevertheless, due to the temperature limitations of the SC-CO_2_ extraction technique, excessive water content may result in dissolving large amounts of nicotine into the water, and the nicotine will not be able to be released with the evaporation of the entrainer. During the experiment, samples infiltrated by the entrainer with a lower volume fraction of ethanol still remained wet after 120 min or more of supercritical fluid extraction. In the case of a small amount of target extract, we do not consider the resistance caused by solubility, and the mass transfer law is usually explained by the hot-ball model [34]. In this model, CO_2_ is adsorbed on the surface of the solute particles before diffusing into the interior of the solute. The large amount of cellulose in tobacco roots will retain nicotine through hydrogen bonding [25]. If we add a suitable entrainer, CO_2_ will diffuse more easily into the tobacco root particles, and at the same time, the retention effect of hydrogen bonds on nicotine will be greatly reduced in the presence of ethanol and water. This also explains why 90% ethanol can significantly improve the yield of nicotine. With the above experimental design and optimisation of conditions, we improved the yield from 0.22% to about 0.49%. The enhancement was about 122.7%.

Due to the high extraction efficiency of SC-CO_2_ technology, the purity of nicotine in the crude extract was already about 60%. In addition to nicotine, the crude tobacco rhizome extract also contained palmitic acid, oleic acid, and oleic acid derivatives [8,13]. This is different from tobacco leaves extract, which contains other active plant substances, such as solanesol, rutin, and so on. From Figure 3, we can find that the retention time of the vast majority of impurities in the crude extract is higher than that of nicotine. This means that in the future, the impurities in the extract can be purified industrially by the difference between their boiling point and that of nicotine. The entrainer used in this experiment was ethanol due to safety and manipulability considerations under laboratory conditions. It may be possible to choose a more suitable entrainer in future studies. In this way, a lower amount of entrainer could be used to reduce its effect on the purification.

## 4. Materials and Methods

### 4.1. Chemicals

We used 99.9% CO_2_ (Dumaoai Purification gas Co., Ltd., Shanghai, China). Nicotine standard (99% purity, 4000 mg/L) was obtained from Anpel-trace standard Technical Service Co., Ltd. (Shanghai, China). Other chemical reagents used in the experiment were analytically pure.

### 4.2. Tobacco Materials

The tobacco rhizomes used for the experiment were purchased in March 2023 from a local tobacconist in Zimbabwe. The rhizomes obtained were washed with water to remove dust and soil. Then, they were dried in an oven at 80 °C for 12 h. After that, the tobacco rhizomes were ground through a pulveriser until they could pass through a 40–60 mesh sieve. The resulting rhizome powder was dispersed and macerated using appropriate proportions of ethanol before being extracted by SC-CO_2_ (Jiangsu gaoke Pharmaceutical Equipment Co., Ltd., Nantong, China). The rhizome cells were crushed by an ultrasonic crusher (Xinzhi Biotechnology Co., Ltd., Ningbo, China) for about 10 mins.

### 4.3. Extraction Process

The solid–liquid mixture of pre-treated samples was added into the SC-CO_2_ extraction apparatus, and the parameters of the apparatus were adjusted to set the extraction pressure to 25 MPa, the temperature to 45–65 °C, the separation pressure to 4.5–6 MPa, and the valve frequency of the CO_2_ cylinder to 16 KHz. The length of the whole extraction process was 120 min. The fraction in the separator kettle was collected to obtain the tobacco rhizome extract after completion of the extraction. In addition to the experimental factors focused on in this study, the extraction time and pressure, which were not the key factors for this study, used in the experiments were summarised from previous studies [13,25,32,35]. The range of extraction time for this study was typically 60–120 min, depending on the instrument size and sample quality. Additionally, the positive impact of time on yield tends to plateau as it increases. In some experiments, five times the volume entrainer was utilised; however, if the extraction time is too short, complete evaporation may not occur. A similar situation arises with pressure selection, where the usual range for extraction pressure falls between 8 and 25 MPa. To eliminate any influence from time and pressure on nicotine yield in our study, we opted for longer periods of time and higher pressures in each experiment.

**Figure 4:**
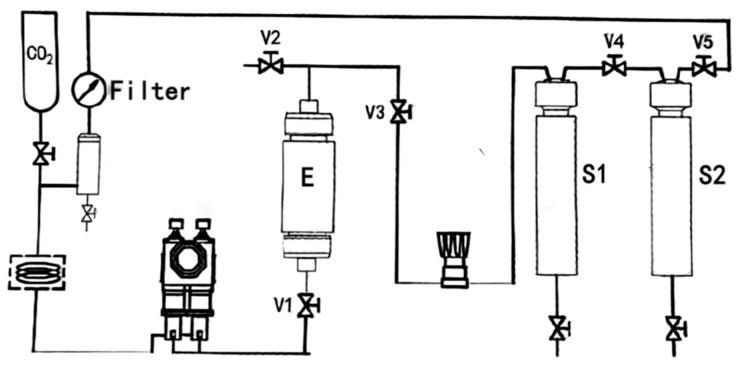
The schematic diagram of the supercritical CO_2_ unit.

A schematic diagram of the supercritical CO_2_ unit used for extraction is shown in Figure 4. The sample was placed into the extraction kettle (E) and then pressurised and heated. The evaporated crude extract was then condensed and refluxed to the separation kettle (S1, S2).

### 4.4. GC-MS Analysis

Hexane was used as a solvent for this analysis. Complete GS-MS analysis was performed using a gas chromatography–mass spectrometer (Thermo Trace ISQ, Waltham, MA, USA). The column was an HP-Wax quartz capillary column (30 m × 0.25 mm, 0.25 μm). Inlet temperature was 230 °C, and the carrier gas was He (>99.999%). The flow rate of the gas phase was 1.0 mL/min. The HP-Wax quartz capillary column was programmed to start from 100 °C and hold for 2 min. The temperature was then increased to 250 °C at a rate of 30 °C/min and kept isothermal for 15 min. The injecting mode was split-flow injection at a ratio of 20:1. The injection volume was 1 μL. The ionisation mode was EI, and the ionisation energy was 70 eV. The ion source temperature was 230 °C, the same as the transfer line temperature. The solvent delay time was 2 min and the scanning range was 41–300 amu. The mass spectrometry standard library used was NIST.

Relative percentage content was calculated from the GC peak area using the normalisation method. The method used for quantitative analysis was a standard curve. Three different concentrations of nicotine were made by dissolving nicotine standards in hexane. The R^2^ of the calibration curve was 0.997. All analyses were performed in triplicate.

### 4.5. Purification

Firstly, the resulting suspension of crude extract of tobacco rhizomes was concentrated by rotary evaporation at 60 °C for 30 min, and then rotary evaporation was carried out at 30 °C for 1 h. Secondly, silica gel powder was added and rotary until dry to obtain a mixed dry sample of nicotine extract with silica gel. The paste sample was obtained by infiltrating with 10 times the volume of triethylamine. Thirdly, it was eluted with a mixture of methanol and dichloromethane at a volume ratio of 1:10. Finally, the purified nicotine product was obtained, and its purity was about 98%.

## 5. Conclusions

To date, methods for the treatment of tobacco waste are still being developed worldwide. This experiment provides a method to obtain nicotine from tobacco rhizomes. The aim is to increase the economic value of tobacco waste while reducing its pollution and harm to the environment. Moreover, the vast majority of harmful substances in tobacco rhizomes have been removed after extraction by SC-CO_2_. In the future, these extracted materials can also be used as organic fertiliser.

In this study, we investigated the optimal conditions for the extraction of nicotine from tobacco rhizomes by SC-CO_2_. The results showed that the yield was greatly improved after applying the experimental design and improvements. Also, the nicotine purity of the crude extract is at a high level, which has a very good purification potential for future industrialisation.

## Figures and Tables

**Table 1:** The orthogonal experiments’ design and results for total nicotine extraction yield (%) of fine tobacco rhizomes for extremum difference analysis.

No.	Temperature	Ratio	Ethanol Fraction of Entrainer	Yield
1	45	1:1	80	0.26%
2	45	1:3	90	0.29%
3	45	1:5	100	0.20%
4	55	1:1	90	0.21%
5	55	1:3	100	0.30%
6	55	1:5	80	0.27%
7	65	1:1	100	0.22%
8	65	1:3	80	0.24%
9	65	1:5	90	0.49%
10	65	1:5	90	0.48%
11	65	1:5	90	0.49%

**Table 2:** The extremum difference analysis results for three factors at three different levels of the orthogonal experiments for the total nicotine extraction yield (%) of fine tobacco rhizomes.

Term	Level	Temperature	Ration	Volume Fraction
K-Value	1	0.74	0.69	0.77
	2	0.78	0.83	0.99
	3	0.95	0.95	0.71
K avg-Value	1	0.25	0.23	0.26
	2	0.26	0.28	0.33
	3	0.32	0.32	0.24
Best level		3	3	2
R		0.07	0.09	0.09
Account of Level		3	3	3
Number of repetitions per level (r)		3	3	3

## Data Availability

Data are contained within the article.
